# Supplementary material for: Altered succinylation of mitochondrial proteins, APP and tau in Alzheimer’s disease
Source: Nat Commun. 2022 Jan 10;13:159. doi: 10.1038/s41467-021-27572-2 (PMC8748865; doi:10.1038/s41467-021-27572-2)
Supplement: Supplementary file 1 — Supplementary Information [file 41467_2021_27572_MOESM1_ESM.pdf]

Supplementary Information

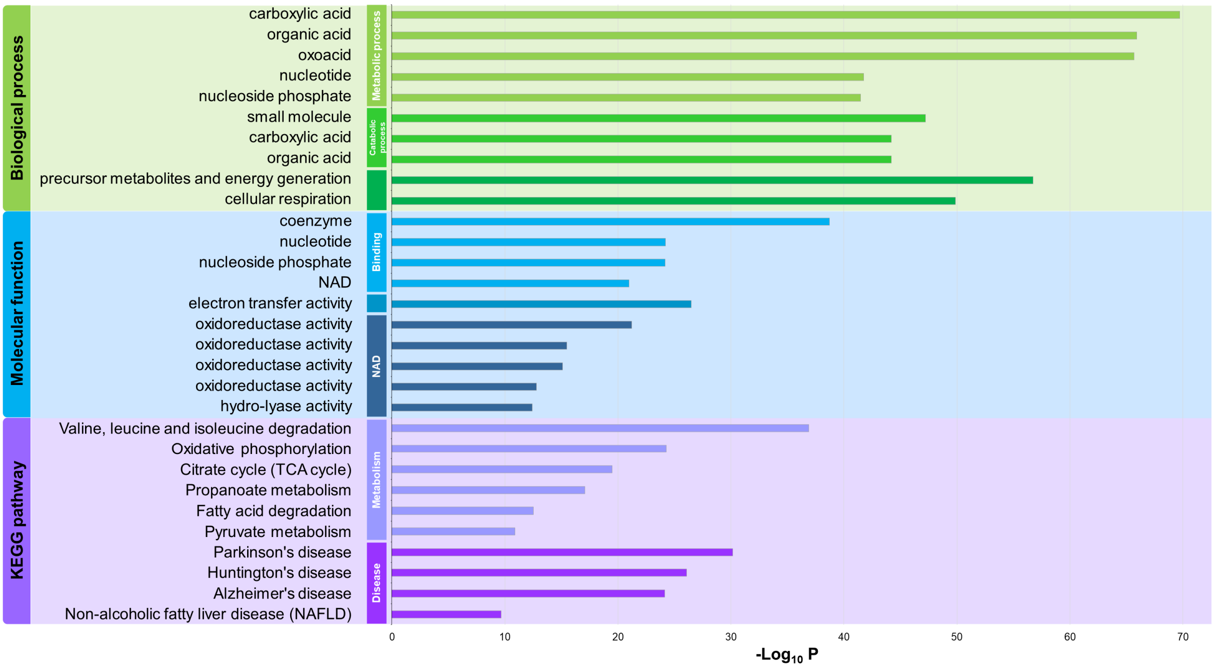

Supplementary Figure 1. Gene ontology functional analysis of human brain succinylome. The graph shows *p*-values (step-down Bonferroni correction) for the most significant specific terms reflecting biological process (green field), molecular function (blue field) and cell component (purple field) (**Supplementary Data 3** for detail).

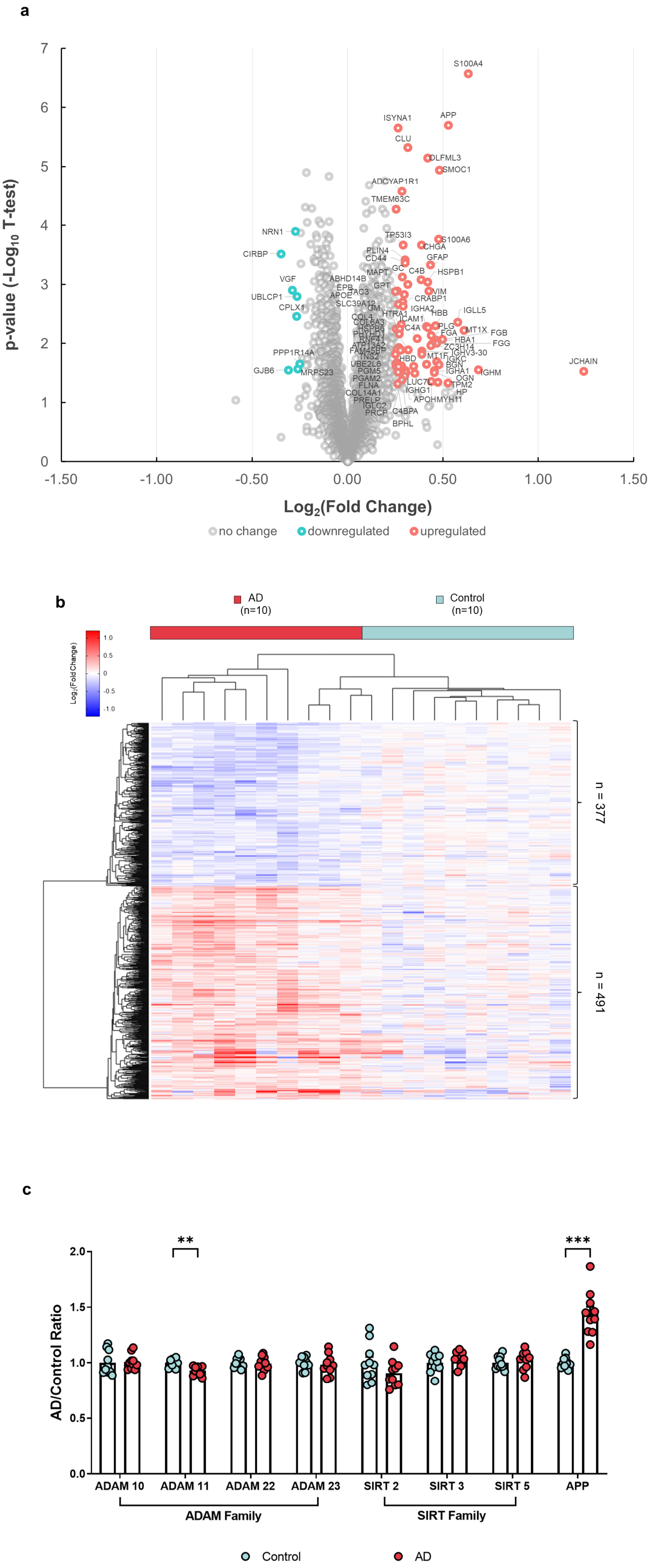

Supplementary Figure 2. Comparison of the brain global proteomics from ten controls and ten patients with AD reveal many specific differences.

a. Volcano plot of global proteomic results comparing brains from controls and AD patients. The signal detection result shows the magnitude (mean expression difference, x-axis) and significance ($-\log_{10}$ p-value, y-axis) for brain protein level changes associations of AD. Each spot represents a specific protein. Green symbols indicate proteins that decline significantly while red symbols indicate proteins that are elevated significantly in AD brains (p<0.05, $|\log_2 FC|$>0.25).
b. Supervised hierarchical clustering of the 868 proteins whose levels differ (p<0.05) between AD and control.
c. Proteomic analysis indicates that the protein levels of the α-secretase (ADAM10) are not altered in AD. The data are shown as the mean with error bars from SEM (n = 10, ***: $p < 0.001$, **: $p < 0.01$, two-way ANOVA followed by Bonferroni's multiple comparisons test).

a

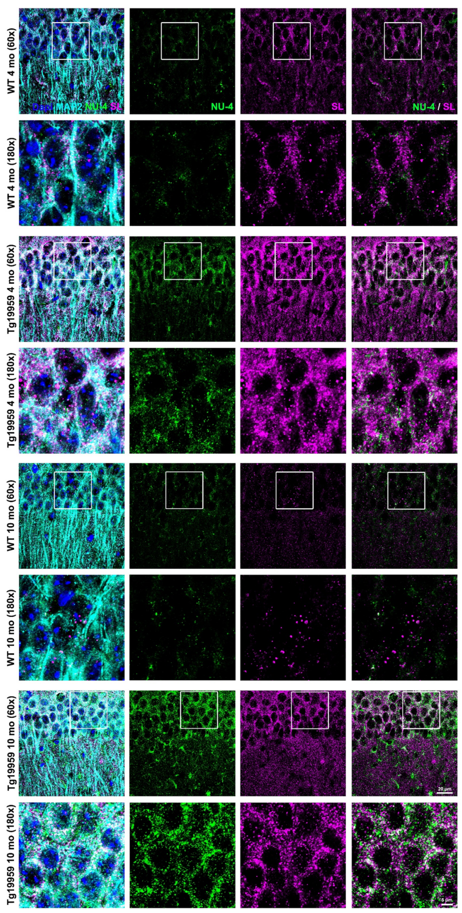

b

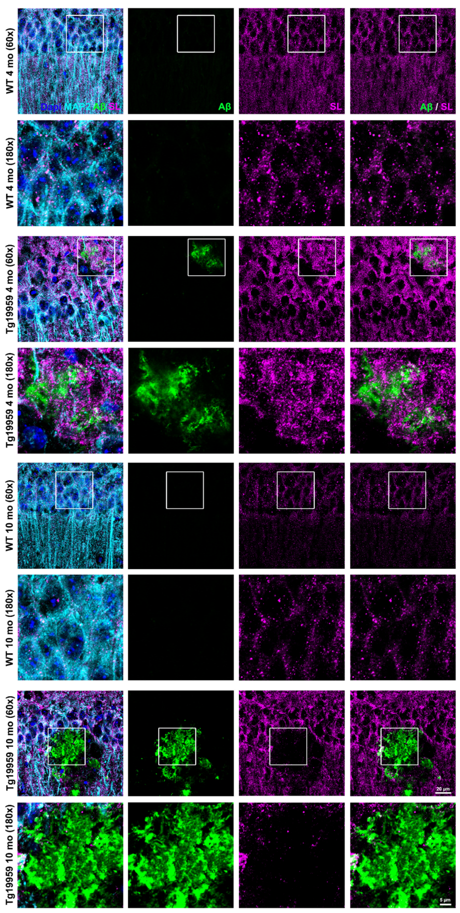

c

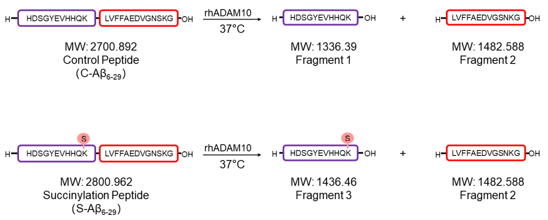

d

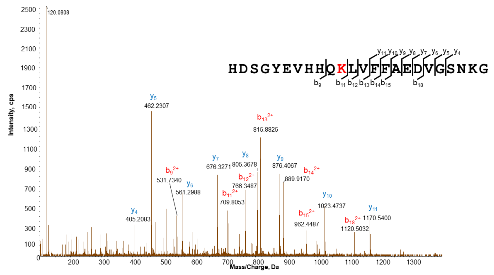

e

Peptide	Sequence	Residues	Quality
C-Aβ6-29	HDSGYEVHHQKLVFFAEDVGNSKG	24	>95%
S-Aβ6-29	HDSGYEVHHQ{Lys-Succinylation}LVFFAEDVGNSKG	24	

f

Compounds	Retention time (min)	Precursor ion (m/z)	Compounds parameter		Fragment ion
			Declustering potential (V)	Collision energy (V)	
Peptide 1	5.64	675.826	50	30	876.406
Peptide 2	5.71	700.83	50	30	815.884
Fragment 1	1.2	668.805	50	40	788.321
Fragment 2	6.21	691.851	50	30	876.406
Fragment 3	3.87	479.545	50	25	560.21

g

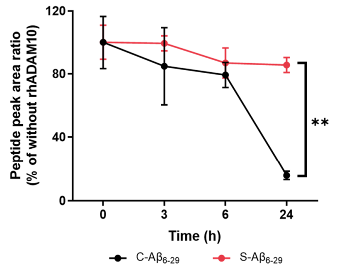

Supplementary Figure 3. Inhibition of succinylated K612 on $A\beta_{6\text{-}29}$ in the α-cleavage assay and succinylated $A\beta_{42}$ using succinyl-CoA *in vitro* and its effect on ThT fluorescence assay.

a and **b.** High resolution confocal laser microscopy images depicting the co-localization of succinylation and Aβ pathology in hippocampal CA1 sections from 4-month-old and 10-month-old Tg19959 or WT mice. NU-4 or Aβ plaques (green), pan-succinyl-lysine (magenta), MAP2 (cyan), and DAPI (dark blue).

c. The schematic diagram of α-cleavage assay for peptides.

d. The MS/MS spectra of the synthetic succinylated $A\beta_{6\text{-}29}$ peptide used in assay (Succinylation lysine residue is highlighted in red text).

e. Properties of $A\beta_{6\text{-}29}$ peptides used in the α-cleavage MRM assay.

f. Multiple Reaction Monitoring (MRM) parameters used in assay for quantitation with their retention time of targeted peptides and their fragments.

g. The control $A\beta_{42}$ peptide and succinylated $A\beta_{42}$ peptide quantitation in the α-cleavage assay. Peptide peak area ratio values were calculated and were shown relative to corresponding controls without rhADAM10. Each sample was run in triplicate (except the 6 h samples were run in duplicate). The data are shown as the mean with error bars from SEM (**: $p < 0.01$, two-way ANOVA followed by Bonferroni's multiple comparisons test).

a

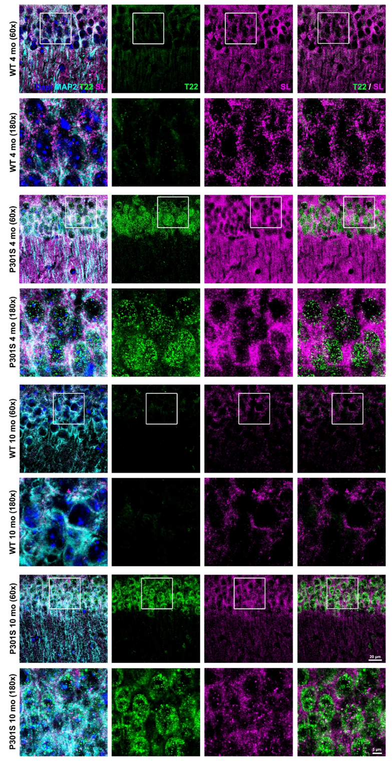

b

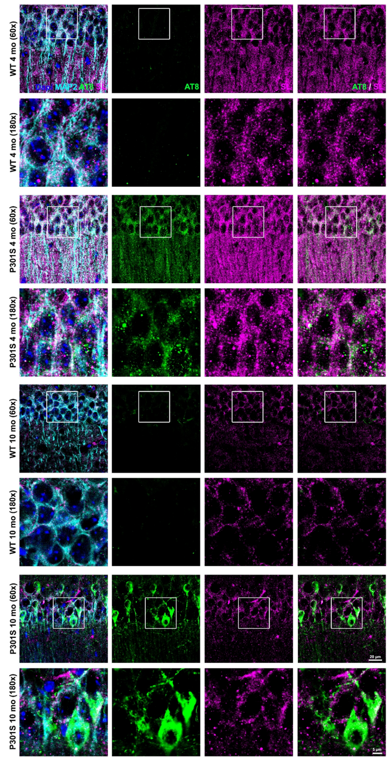

c

Peptide	Sequence	Residues	Quality
PHF6	Ac-VQIVYK-NH_2	6	
S-PHF6	Ac-VQIVY{Lys-Succinylation}-NH_2	6	
PHF6*	Ac-VQIINK-NH_2	6	
A-PHF6*	Ac-VQIIN{Lys-Acetylation}-NH_2	6	>95%
tau (296-321)	Ac-NIKHVPGGGSVQIVYKPVDLSKVTSK-NH_2	26	
S-tau (296-321)	Ac-NIKHVPGGGSVQIVY{Lys-Succinylation}PVDLSKVTSK-NH_2	26	

d

244 (M)QTAPVPMPDLK NVKSKIGSTENLKHQPGGGK R1
306 VQIVYKPVDLS KVTSKCGSLGNIHHKPGGGQ R3
337 VEVKSEKLDFKDRVQSKIGSLDNITHVPGGGN R4
369 KKIE R′

e

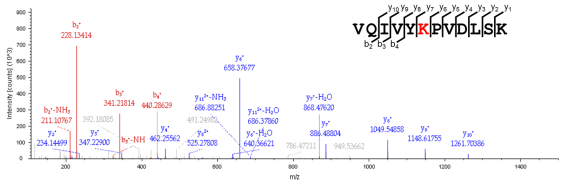

f

SequencePercolator	Modifications	χCorr	Percolator PEP
VQIVYKPVDLSK	K311	3.07	0.011
IGSTENLKHQPGGGK	K267	4.72	0.0068
SKIGSTENLK	K259	2.45	0.062
SEKLDFKDR	K343, K347	2.52	0.12
VQIVYKPVDLSKVTSK	K317, K321	3.06	0.032
SKIGSTENLKHQPGGGK	K259, K267	3.92	0.015
HQPGGGKVQIVYKPVDLSK	K274, K311	4.98	0.003
CGSLGNIHHKPGGGQVEVK	K331	4.91	0.0036
MQTAPVPMPDLKNVKSK	K257	2.98	0.071

g

244 (M)QTAPVPMPDLK NVKSKIGSTENLKHQPGGGK R1
306 VQIVYKPVDLS KVTSKCGSLGNIHHKPGGGQ R3
337 VEVKSEKLDFKDRVQSKIGSLDNITHVPGGGN R4
369 KKIE R′

h

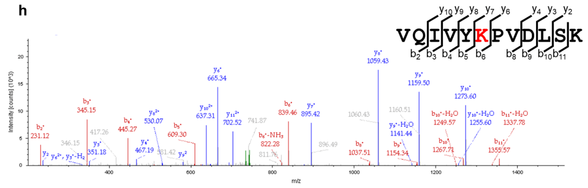

i

SequencePercolator	Modifications	χCorr	Percolator PEP
VQSKIGSLDNITHVPGGGNK	K353	3.76	0.029
VQIVYKPVDLSKVTSK	K321	3.58	0.0040
VQIVYKPVDLSK	K311	3.68	0.0095
VTSKCGSLGNIHHKPGGGQVEVK	K321	2.84	0.052
IGSTENLKHQPGGGK	K267	2.66	0.024
CGSLGNIHHKPGGGQVEVK	K332	3.36	0.025
SKIGSTENLK	K259	2.95	0.077
SEKLDFKDR	K347	3.06	0.22

j

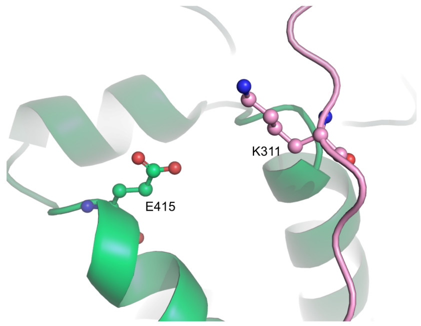

Supplementary Figure 4. Characterization of succinylated K19 and ^{15}N K19 using succinyl-CoA *in vitro*.

a and **b.** Confocal fluorescence micrographs showing succinylation and tau pathology in the hippocampal CA1 region of 4-month-old and 10-month-old TgP301S or WT mice. Brain sections were stained using antibodies against T22 or AT8 (green), pan-succinyl-lysine (magenta) and MAP2 (cyan), and counterstained with DAPI (dark blue).

c. Properties of peptides used in the self-aggregation assay and STD NMR.

d. MS/MS identified sequence of succ-lysines peptides on K19 following succinylation with Succinyl-CoA *in vitro*. Residue numbering is based on the numbering of the longest tau isoform, htau40 (441 residues), and skips directly from residue 274 to 305 aa as a result of the absence of the second repeat (residues 275-305 aa). Formatting is used as follows: red, lysines (K) with succinyl group; green box, sequence covered by MS analysis.

e. MS/MS spectra for identification and quantification of K311 succinylation on K19 following succinylation *in vitro*. b and y ions indicate peptide backbone fragment ions containing the N and C terminal, respectively. $^{2+}$ indicates doubly charged ions. Succ-Lysine is colored in red.

f. K19 succinylation peptides identified by MS/MS (χCorr ≥ 2.11).

g. MS/MS identified sequence of succ-lysines peptides on ^{15}N K19 following succinylation with Succinyl-CoA *in vitro*. Residue numbering is based on the numbering of the longest tau isoform, htau40 (441 residues), and skips directly from residue 274 to 305 aa as a result of the absence of the second repeat (residues 275-305 aa). Formatting is used as follows: red, lysines (K) with succinyl group; green box, sequence covered by MS analysis.

h. MS/MS spectra for identification and quantification of K311 succinylation on ^{15}N K19 following succinylation *in vitro*. b and y ions indicate peptide backbone fragment ions containing the N and C terminal, respectively. $^{2+}$ indicates doubly charged ions. Succ-Lysine is colored in red.

i. ^{15}N K19 succinylation peptides identified by MS/MS (χCorr ≥ 2.11).

j. Three-dimensional structure of K311 on K19 and E415 on α-tubulin during the tau-tubulin interactions.
