# Supplementary material for: Altered succinylation of mitochondrial proteins, APP and tau in Alzheimer’s disease
Source: Nat Commun. 2022 Jan 10;13:159. doi: 10.1038/s41467-021-27572-2 (PMC8748865; doi:10.1038/s41467-021-27572-2)
Supplement: Supplementary file 3 — Description of Additional Supplementary Files [file 41467_2021_27572_MOESM3_ESM.pdf]

Description of Additional Supplementary Files

File name: Supplementary Data 1
Description: Criteria for the individual patients who donated the autopsied brains. All samples were from Broca's area (BM-44/45, frontal lobe).

File name: Supplementary Data 2
Description: Subcellular distribution of 314 succinylated proteins with the number of succinylated site in human brains, related to Figure 2a and 2c. 21 proteins with over 10 independent succinylated lysines are labeled in red.

File name: Supplementary Data 3
Description: Biological process, molecular function and KEGG pathway enrichment analysis of succinylated proteins according to the classification of GO annotation (p-values computed by a two-sided hypergeometric test, with step-down Bonferroni correction, $p<0.01$ and gene number>5), related to Supplementary Figure 1.

File name: Supplementary Data 4
Description: Succinylation proteomics data from AD and control human brains, related to Figure 3a. 29 peptides with significant differences ($p<0.05$) in succinylation between control and AD brains are labeled in red.

File name: Supplementary Data 5
Description: Succinylation and global proteomics data of 29 peptides (from 20 proteins) differs ($p < 0.05$) in the AD and controls with subcellular distribution.

File name: Supplementary Data 6
Description: Succinylation and global proteomics data of 29 peptides (from 20 proteins) differs ($p < 0.05$) in the AD and controls with subcellular distribution.

File name: Supplementary Data 7
Description: The succinylation proteomics database search information used in the paper.
