# Supplementary material for: Altered succinylation of mitochondrial proteins, APP and tau in Alzheimer’s disease
Source: Nat Commun. 2022 Jan 10;13:159. doi: 10.1038/s41467-021-27572-2 (PMC8748865; doi:10.1038/s41467-021-27572-2)
Supplement: Supplementary file 4 — Supplementary Data 1 [file 41467_2021_27572_MOESM4_ESM.pdf]

Supplementary Data 1. Criteria for the individual patients who donated the autopsied brains.
All samples were from Broca's area (BM-44/45, frontal lobe).

Normals								
Donor	Sample ID	cohort	Sex	Age	PMI (min)	CDR	Mean Plaques (mm2)	Braak Score
1	80679	1st	M	70	606	1	0	0
2	80680		F	69	757	0.5	0	0
3	80688		M	78	485	0	0	2
4	80695		F	68	1155	0.5	0	0
5	80696		M	69	225	0.5	0	0
6	91230	2nd	M	64	513	0	0	0
7	91234		F	75	195	0.5	0	3
8	91238		M	78	355	0	0	0
9	91250		M	79	545	0	0	0
10	91254		M	75	998	0	0	0
Mean				72.5	583	0.3	0	0.5
SEM				1.6	99	0.1	0.0	0.3

AD patients								
Donor	Sample ID	cohort	Sex	Age	PMI (min)	CDR	Mean Plaques (mm2)	Braak Score
1	80664	1st	M	78	455	2	10.85	4
2	80671		F	79	390	3	20.48	6
3	80674		M	74	890	3	12.79	6
4	80687		M	69	325	3	28.59	6
5	80703		M	75	480	2	22.47	6
6	91226	2nd	M	75	883	3	18.88	6
7	91242		M	79	480	3	10.46	6
8	91246		F	75	285	1	24.15	6
9	91258		M	78	310	2	12.58	6
10	91262		M	64	430	3	27.21	6
Mean				74.6	493	2.5	18.8	5.8
SEM				1.5	69	0.2	2.2	0.2
