# Supplementary material for: Altered succinylation of mitochondrial proteins, APP and tau in Alzheimer’s disease
Source: Nat Commun. 2022 Jan 10;13:159. doi: 10.1038/s41467-021-27572-2 (PMC8748865; doi:10.1038/s41467-021-27572-2)
Supplement: Supplementary file 11 — Reporting Summary [file 41467_2021_27572_MOESM11_ESM.pdf]

nature research

Corresponding author(s): Gary E. Gibson

Last updated by author(s): Nov 2, 2021

Reporting Summary

Nature Research wishes to improve the reproducibility of the work that we publish. This form provides structure for consistency and transparency in reporting. For further information on Nature Research policies, seeAuthors & Referees and theEditorial Policy Checklist .

Statistics

For all statistical analyses, confirm that the following items are present in the figure legend, table legend, main text, or Methods section.

n/a	Confirmed	
	x	The exact sample size (*n*) for each experimental group/condition, given as a discrete number and unit of measurement
	x	A statement on whether measurements were taken from distinct samples or whether the same sample was measured repeatedly
	x	The statistical test(s) used AND whether they are one- or two-sided<br>*Only common tests should be described solely by name; describe more complex techniques in the Methods section.*
x		A description of all covariates tested
	x	A description of any assumptions or corrections, such as tests of normality and adjustment for multiple comparisons
	x	A full description of the statistical parameters including central tendency (e.g. means) or other basic estimates (e.g. regression coefficient) AND variation (e.g. standard deviation) or associated estimates of uncertainty (e.g. confidence intervals)
	x	For null hypothesis testing, the test statistic (e.g. *F*, *t*, *r*) with confidence intervals, effect sizes, degrees of freedom and *P* value noted<br>*Give P values as exact values whenever suitable.*
x		For Bayesian analysis, information on the choice of priors and Markov chain Monte Carlo settings
x		For hierarchical and complex designs, identification of the appropriate level for tests and full reporting of outcomes
x		Estimates of effect sizes (e.g. Cohen's *d*, Pearson's *r*), indicating how they were calculated

Our web collection on statistics for biologists contains articles on many of the points above.

Software and code

Policy information about availability of computer code

Data collection	Xcalibur 3.0 operation software and Orbitrap Fusion Tune 2.0 (Thermo Scientific Inc.) was used for data acquisition of global proteomics fractions and enriched SuccK samples.<br>Sciex OS 1.3 software was used for MRM HR data acquisition of C-Aβ6-29 peptide, S-Aβ6-29 peptide, and 15N labeled K19.<br>Bruker's Topspin software (version 3.2) was used for NMR spectrum acquisition.
Data analysis	Proteome Discoverer 2.2 (PD2.2, Thermo Scientific Inc.) was used to extract relevant MS/MS spectra from the raw file, determine the precursor charge state and the quality of the fragmentation spectrum and identify proteins. Percolator algorithm in percolator node is part of processing workflow in PD 2.2 software. (Percolator is a superior validation algorithm that uses a machine learning approach that requires a sufficient number of target and decoy matches to calculate the FDRs for peptide-spectrum matches (PSMs) and for peptide groups.)<br>Perseus software (version 1.6.0.7) was used for statistical analysis of the peptide and protein abundance data. The search result including ratio, p-value, succinylated peptide abundance for each sample was output to Microsoft Excel software (MicroSoft Office 365) for further data analysis.<br>Cytoscape (version 3.6.1) and stringAPP (version 1.4.0) software were used to identify the subcellular localization of the identified candidates, and visualization was implemented in FunRich (version 3.1.3).<br>The web-based Motif-X program (version 1.2 10.05.06) (http://motif-x.med.harvard.edu/) was used to identify statistically significant motifs from the large post-translational modification peptide sequences.<br>IceLogo tool (https://iomics.ugent.be/icelogoserver/) was used to create the heat map of 15 amino acid compositions.<br>ClueGO (version 2.5.1) was used to analysis and visualization of Gene Ontology terms associated with succinylated proteins.<br>Cluster (version 3.0) was used to cluster the global proteomic proteins data and Java TreeView3.0 beta01 (https://bitbucket.org/TreeView3Dev/treeview3/) was used to visualize the clustering results.<br>Image Studio Lite (version 5.2) was used for the quantification of immunoblotting analyses, and all statistical analysis and visualizations were implemented in Graphpad Prism 8 (GraphPad Software, San Diego, CA, USA).<br>Fiji software (Fiji, RRID:SCR_002285) was used for the quantification of cellular immunocytochemistry, the results were analyzed with

SPSS, and visualized in Graphpad Prism 8 (GraphPad Software, San Diego, CA, USA).
Confocal fluorescence images were acquired using the ZEN black edition (version 2.3; Carl Zeiss) software. ImageJ-Fiji (version 15.2, NIH) software was for quantification of in vivo immunofluorescence, and all statistical analysis and visualizations were implemented in Graphpad Prism 8 (GraphPad Software, San Diego, CA, USA).
ImageJ (version 1.52a) was used for the quantification of the width and height of the fiber helix, all statistical analysis and visualization was implemented in Graphpad Prism 8 (GraphPad Software, San Diego, CA, USA).
Graphpad Prism 8 (GraphPad Software, San Diego, CA, USA) was used for other statistical analysis and visualizations.

For manuscripts utilizing custom algorithms or software that are central to the research but not yet described in published literature, software must be made available to editors/reviewers. We strongly encourage code deposition in a community repository (e.g. GitHub). See the Nature Research guidelines for submitting code & software for further information.

Data

Policy information about availability of data

All manuscripts must include a data availability statement. This statement should provide the following information, where applicable:

- Accession codes, unique identifiers, or web links for publicly available datasets
- A list of figures that have associated raw data
- A description of any restrictions on data availability

All data needed to evaluate the conclusions of this study are available in the manuscript, in the supplementary information files or from the data bases listed in the manuscript. Source data are provided with this paper.
The mass spectrometry proteomics data files including raw MS files, peak list files and search results files were deposited to PRIDE database by ProteomeXchange (PXD015124).
Other data are available from the corresponding author upon reasonable request.

Field-specific reporting

Please select the one below that is the best fit for your research. If you are not sure, read the appropriate sections before making your selection.

☒ Life sciences ☐ Behavioural & social sciences ☐ Ecological, evolutionary & environmental sciences

For a reference copy of the document with all sections, see nature.com/documents/nr-reporting-summary-flat.pdf

Life sciences study design

All studies must disclose on these points even when the disclosure is negative.

Sample size

This study included a total of n=20 (10 AD and 10 NL) samples from 20 unique patients.
Sample size in this discovery study was determined based on our initial proteomics and succinylomics results in the first cohort (5 controls and 5 AD cases) experiment that identified a significant difference of about two dozen of succinylated peptides in AD cases following Student's t test and other statistical analyses.
Based on literature on “Statistical Design for Biospecimen Cohort Size in Proteomics based Biomarker Discovery and Verification Studies” (DOI: 10.1021/pr400132j) and the brain tissue samples with additional enrichment workflow used in our workflow, we decide to add the second cohort analysis to 1) test set-to-set reproducibility and 2) increase sample size to a total of 10 controls and 10 AD cases, that would allow us for achieving greater than 80% probability of reaching verification for discovery of anticipated 20 candidate biomarkers that were present in at least 80% of the cases, a cutoff being used in our study. Thus, as a discovery stage of studies, we believe sample size chosen in this work is sufficient.
With regard to in vivo experiment no statistical methods were used to pre-determine sample sizes, but our sample sizes are similar to those reported in previous publications on mouse models of Alzheimer’s Disease (see below).
1. Tg19959 mice
Pre-plaque conformational changes in Alzheimer’s disease-linked Aβ and APP (DOI: 10.1038/ncomms14726)
Detection of Aβ plaque-associated astrogliosis in Alzheimer's disease brain by spectroscopic imaging and immunohistochemistry (DOI: 10.1039/C7AN01747B)
2. P301S mice
Tau-positive nuclear indentations in P301S tauopathy mice (DOI: 10.1111/bpa.12407)
Long-term treadmill exercise attenuates tau pathology in P301S tau transgenic mice (DOI: 10.1186/1750-1326-9-54)

Data exclusions

No samples or animals were excluded from the analysis except one staining result in 10 m.o. TG19959 mice. This analysis was performed exclusion of outliers using Grubbs' test (Alpha = 0.01).
No any MS data files and results were excluded.

Replication

Proteomics and succinylome replication were achieved through validation in two cohorts from independent batches of brains.
At least two independent biological repeats were performed, except the negative-staining electron microscopy. All cell culture experiments presented in the manuscript were repeated at least three times independently with similar results.
We specified the number of biological replicates and independent experiments in the respective figure legends.

Randomization

The human autopsy brains samples were all from very similar patients as determined by plaques, tangles and Braak neuritic pathology score. Thus, they randomly allocated into groups.
Proteomics sample preparation and labeling, and succinylomics sample preparation were randomized. All samples were analyzed in randomized order for label free quantitative analysis of succinylomes.

The cells for immunocytochemistry studies within particular brain regions were randomly selected. The mice were randomized and put into separate/groups cages for experiments.

Blinding

Investigators were blinded to clinical histopathology assessments during sample selection. Human specimens were de-identified and assigned a unique numerical code. Researchers responsible for data generation were blinded and had no access to basic information about donors (age, sex, group allocations).
For experiments other than those involving human specimens, similar grouping information was unavailable to researchers involved in data generation and analysis.

Reporting for specific materials, systems and methods

We require information from authors about some types of materials, experimental systems and methods used in many studies. Here, indicate whether each material, system or method listed is relevant to your study. If you are not sure if a list item applies to your research, read the appropriate section before selecting a response.

Materials & experimental systems

n/a	Involved in the study	
☐	☒	Antibodies
☐	☒	Eukaryotic cell lines
☒	☐	Palaeontology
☐	☒	Animals and other organisms
☐	☒	Human research participants
☒	☐	Clinical data

Methods

n/a	Involved in the study	
☒	☐	ChIP-seq
☒	☐	Flow cytometry
☒	☐	MRI-based neuroimaging

Antibodies

Antibodies used

Pan anti-succinyllysine (1:200 for immunoprecipitation; 1:1,000 for western blot; 1:2,000 for immunofluorescence; Cat # PTM-401, PTM Biolabs Inc., Chicago, IL, USA);
anti-DLST (1:600; Cat # 11954, Cell Signaling Technology, Inc., Danvers, MA, USA);
mouse anti-Cox IV (1:1,000; Cat # 11967, Cell Signaling Technology, Inc., Danvers, MA, USA);
β-Actin (13E5) Rabbit antibody (1:1,000; Cat # 4970, Cell Signaling Technology, Inc., Danvers, MA, USA);
s680RD Goat anti-Rabbit IgG Secondary Antibody (1:10,000; Cat # 926-68071, LI-COR Biosciences, Lincoln, NE, USA);
800CW Goat anti-Mouse IgG Secondary Antibody (1:10,000; Cat # 926-32210, LI-COR Biosciences, Lincoln, NE, USA);
Donkey anti-Mouse IgG (H+L) Highly Cross-Adsorbed Secondary Antibody, Alexa Fluor 488 (1:1,000; Cat # A21202, Thermo Fisher Scientific, Waltham, MA, USA);
Donkey anti-Rabbit IgG (H+L) Highly Cross-Adsorbed Secondary Antibody, Alexa Fluor 568 (1:1,000; Cat # A10042, Thermo Fisher Scientific, Waltham, MA, USA);
Anti-MAP2 Antibody (1:2000; Cat # AB15452, Millipore, Burlington, MA, United States);
Cy3 AffiniPure Donkey Anti-Rabbit IgG (H+L) (1:500; Cat # 711-165-152, Jackson ImmunoResearch, West Grove, PA, USA);
647 AffiniPure Donkey Anti-Chicken IgY (IgG) (H+L) (1:500; Cat # 703-005-155, Jackson ImmunoResearch, West Grove, PA, USA);
Anti-Pyruvate dehydrogenase E2/E3bp antibody (1:1000; Cat # ab110333, Abcam, Cambridge, MA, USA);
Pyruvate Dehydrogenase Antibody (1:500; Cat # 2784, Cell Signaling Technology, Inc., Danvers, MA, USA);
Anti-beta Actin antibody (1:4000; Cat # ab8226, Abcam, Cambridge, MA, USA);
COX IV (3E11) Rabbit monoclonal antibody (1:3000; Cat # 4850, Cell Signaling Technology, Inc., Danvers, MA, USA);
Anti-mouse β-Amyloid (1:2,000; Cat #15126S, Cell Signaling Technology, Inc., Danvers, MA, USA);
Anti-Phospho-Tau AT8 (1:2,000; Cat # MN1020, Thermo Fisher Scientific, Waltham, MA, USA);
anti-β-Amyloid 6E10 (1:1,000; Cat # 803001, BioLegend, San Diego, CA, USA);
Anti-KGDHC E1k (1:1,000; Rockland Antibodies and Assays, Limerick, PA, USA);
Anti-KGDHC E2k (1:1,000; Rockland Antibodies and Assays, Limerick, PA, USA);
Anti-KGDHC E3 (Lipoamide Dehydrogenase Antibody) (1:2,000; Cat # 200-4160S, Rockland Antibodies and Assays, Limerick, PA, USA);
Anti-mouse NU-2 (1:4,000; Klein's lab);
Anti-mouse NU-4 (1:2,000; Klein's lab);
Anti-mouse T22 (1:2,000; Kayed's lab).

Validation

All the antibodies except Anti-KGDHC E1k, E2k, NU-2, NU-4 and T22, used are commercially available. The detailed antibody validation profiles are available on the website of designated companies.
Anti-KGDHC E1k was made by Rockland Antibodies and Assays (Limerick, PA, USA) from the human gene sequence. Immunoreactivity was tested with purified proteins and cells on Westerns (Shi Q, Chen H L, Xu H, et al. Reduction in the E2k subunit of the α-ketoglutarate dehydrogenase complex has effects independent of complex activity[J]. Journal of Biological Chemistry, 2005, 280(12): 10888-10896.).
Anti-KGDHC E2k was made by Rockland Antibodies and Assays (Limerick, PA, USA) from the human gene sequence. Immunoreactivity was tested with purified proteins, tissues from knockdown mice and cells on Westerns (Yang L, Shi Q, Ho D J, et al. Mice deficient in dihydrolipoyl succinyl transferase show increased vulnerability to mitochondrial toxins[J]. Neurobiology of disease, 2009, 36(2): 320-330; Shi Q, Chen H L, Xu H, et al. Reduction in the E2k subunit of the α-ketoglutarate dehydrogenase

complex has effects independent of complex activity[J]. Journal of Biological Chemistry, 2005, 280(12): 10888-10896.).
Anti-mouse NU-2 was reactivity validated by Klein's lab (Lambert M P, Velasco P T, Chang L, et al. Monoclonal antibodies that target pathological assemblies of Aβ[J]. Journal of neurochemistry, 2007, 100(1): 23-35.).
Anti-mouse NU-4 was reactivity validated by Klein's lab (Lambert M P, Velasco P T, Chang L, et al. Monoclonal antibodies that target pathological assemblies of Aβ[J]. Journal of neurochemistry, 2007, 100(1): 23-35.).
Anti-mouse T22 was reactivity validated by Kayed's lab (Lasagna-Reeves C A, Castillo-Carranza D L, Sengupta U, et al. Identification of oligomers at early stages of tau aggregation in Alzheimer's disease[J]. The FASEB Journal, 2012, 26(5): 1946-1959.).

Eukaryotic cell lines

Policy information about cell lines

Cell line source(s)	HEK293T cells (Cat # CRL-3216) was obtained from the American Type Culture Collection (ATCC, Manassas, VA, USA).
Authentication	HEK293T cell lines used in the experiments was authenticated by the Vendor.
Mycoplasma contamination	The cell line routinely tested negative for mycoplasma contamination.
Commonly misidentified lines (See ICLAC register)	No commonly misidentified cell lines were used in this study.

Animals and other organisms

Policy information about studies involving animals; ARRIVE guidelines recommended for reporting animal research

Laboratory animals	All the experiments were carried out in four and ten-month-old transgenic mouse models of AD and appropriate controls. Tg19959 mice (that overexpress a double mutant form of the human amyloid precursor protein) were obtained from Dr. George Carlson (McLaughlin Research Institute, Great Falls, MT, USA). P301S (PS19, that overexpress the human tau gene harboring the P301S mutation) transgenic mice were purchased from the Jackson Laboratory (Bar Harbor, ME, USA). Equal number of males and females were used.
Wild animals	This study did not involve wild animals.
Field-collected samples	There were no field collected samples were used in the study.
Ethics oversight	All procedures were approved by the Animal Care and Use Committee of Weill Cornell Medicine.

Note that full information on the approval of the study protocol must also be provided in the manuscript.

Human research participants

Policy information about studies involving human research participants

Population characteristics	All patient information including diagnosis, clinical dementia rating (CDR), age, sex, post-mortem interval (PMI) disease status and neuropathological diagnostic criteria (including mean plaques, and Braak staging) are detailed in Supplementary Table 1.
Recruitment	No donors were recruited. All brain tissues from Broca's area (BM-44/45, frontal lobe) were from the NIH Neurobiobank and Mount Sinai School of Medicine.
Ethics oversight	All experiments were approved by the Animal Care and Use Committee of Weill Cornell Medicine. All human brains used were obtained from Neurobiobank and Mount Sinai School of Medicine. All brain tissues were procured, stored, and distributed according to applicable state and federal guidelines and regulations involving consent, protection of donor anonymity. The de-identified specimens used in this research were exempt from Institutional Review Boards (IRB) review.

Note that full information on the approval of the study protocol must also be provided in the manuscript.
